# Supplementary material for: RehaBEElitation: the architecture and organization of a serious game to evaluate motor signs in Parkinson’s disease
Source: PeerJ Comput Sci. 2023 Mar 15;9:e1267. doi: 10.7717/peerj-cs.1267 (PMC10280492; doi:10.7717/peerj-cs.1267)
Supplement: Supplemental Information 1 [file peerj-cs-09-1267-s001.docx]

**Attachment 1.** Current studies using serious games for rehabilitation or monitoring of motor signs in individuals with PD.

| **Citation** | **Purpose of the game** | **Movements / Exercises required** | **Interface device** | **Symptom / Characteristic assessed** | **Game usability evaluation** | **Game ar­chi­tectur­e design** |
| --- | --- | --- | --- | --- | --- | --- |
| Leblong et al. 2017 | Upper and lower limbs rehabilitation. | Hip and knee flexion, sit-to-stand, rotation of the trunk, spine extension and stretching. | Beyond Your Motion (BYM). | Balance, gait and bradykinesia. | Not evaluated. | Not presented. |
| Foletto et al. 2017 | Fine motor rehabilitation. | Finger pinch, opposing the thumb to the correct finger and hand opening and closing. | Leap Motion Controller (LMC). | No symptoms assessed. | Game Experience Questionnaire (GEQ). | Not detailed. |
| Silva et al. 2017 | Rehabilitation of postural control, gait and cognition. | Movement of the upper limbs and neck, crouching, jumps, and side and multidirectional steps. | Microsoft Kinect. | Postural control, cognitive and motor function and quality of life. | Not evaluated. | Not presented. |
| Cikajlo et al. 2018 | Upper limbs rehabilitation. | Frontal arm elevation (shoulder flexion) and hand opening and closing. | Microsoft Kinect. | Functional and health status. | Not evaluated. | Not detailed. |
| Avola et al., 2018 | Upper and lower limbs rehabilitation. | Knee raise and finger pinch; march in place and hand open and close; and heel or toe raise and slash with clenched fingers and open hands. | Microsoft Kinect, LMC and Head Mounted Display (HMD). | No symptoms assessed. | Nielsen-Shneiderman heuristics and System Usability Scale (SUS). | Not detailed. |
| Oña et al., 2018 | Upper limbs rehabilitation. | Press a key, touch an object on screen, memorise colours and touch objects in the correct sequence, hand opening and closing, finger pinch and forearm pronation and supination. | LMC. | Handgrip strength and manual dexterity. | Questionnaire for evaluating the usability of games (not specified). | Not detailed. |
| Nuic et al. 2018 | Rehabilitation for gait freezing. | Arm extension and lateral displacements; knee flexion and extension; and trunk lateral displacements, rotation and anteroposterior movements. | Microsoft Kinect. | Gait and balance disorders. | Visual analogue scale for the assessment of game acceptability. | Not presented. |
| Dauvergne et al. 2018 | Upper limbs rehabilitation. | Finger tapping. | Tablet. | Clinical and functional status, rhythmic skills, cognitive impairment and quality of life. | Suitability Evaluation Questionnaire (SEQ). | Not presented. |
| Fernández-González et al. 2019 | Upper limbs rehabilitation. | Press a key, touch an object on the screen, memorise colours and touch objects in the correct sequence, hand opening and closing, finger pinch and forearm pronation and supination. | LMC. | Coordination, speed of movement and dexterity of fine movements. | Client satisfaction questionnaire (CSQ). | Not presented. |
| Shah et al. 2019 | Fine motor rehabilitation. | Hand opening and closing and wrist flexion and extension. | LMC. | Game accessibility, comparing the performance (e.g. final score) of PD patients with healthy individuals. | Not evaluated. | Not presented. |
| Sánchez-Herrera-Baeza et al., 2020 | Upper limbs rehabilitation. | Touch an object on screen, memorise colours and touch objects in the correct sequence, hand opening and closing and forearm pronation and supination. | Oculus Rift 2 and LMC. | Muscle strength, coordination, speed of movement, and fine and gross dexterity. | CSQ. | Not presented. |
| Chen et al. 2020 | Finger motor rehabilitation. | Move the controllers to target on and use finger to press the trigger and shoot. | HTC Vive. | No symptoms assessed. | Semi structured interview and SUS. | Not presented. |
| Oña et al., 2020 | Hand motor rehabilitation. | Hand opening and closing and horizontal shoulder adduction and abduction. | LMC and Oculus Rift. | Manual dexterity. | Satisfaction questionnaire (not specified). | Not detailed. |
| Yuan et al. 2020 | Gait rehabilitation. | Antero-posterior and medial-lateral stepping. | Stepping mat. | Balance and motor coordination. | Not evaluated. | Not presented. |
| Stanica et al. 2020 | Upper and lower limbs rehabilitation. | Shoulder, forearm and ankle flexion and extension; shoulder abduction and adduction; forearm pronation and supination; wrist extension and adduction; spinning wheel; jab punches; hip flexion and abduction; and knee flexion. | HTC Vive and Myo Gesture Control Armband. | Strength, coordination, mobility and effort level. | Questionnaire assessing different aspects related to the use of the system (not specified). | Not detailed. |
| Bevilacqua et al. 2021 | Rehabilitation of balance, functional mobility and gait. | Side steps, arm raising and clapping. | Inter Real Sense camera. | Cognitive impairment, gait performance, balance, motor impairment, mood, physical and mental health, fear of falling, mobility and functional capacity. | Ad hoc scale on satisfaction, Assistive Device Predisposition Assessment (ATDPA) and Unified Theory of Acceptance and Use of Technology (UTAUT). | Not detailed. |
| Cemim et al. 2021 | Upper limbs rehabilitation. | Hand opening and closing, finger tapping, wrist flexion and extension and wrist adduction and abduction. | LMC. | Upper limbs disability, cognitive and motor function, and quality of life. | Semi structured interview. | Not presented. |
| Blanc et al. 2022 | Upper limbs rehabilitation. | Elbow flexion and extension, and shoulder adduction and abduction. | BYM. | No symptoms assessed. | UTAUT. | Not presented. |
