# Supplementary figures and images for: RehaBEElitation: the architecture and organization of a serious game to evaluate motor signs in Parkinson’s disease
Source: PeerJ Comput Sci. 2023 Mar 15;9:e1267. doi: 10.7717/peerj-cs.1267 (PMC10280492; doi:10.7717/peerj-cs.1267)

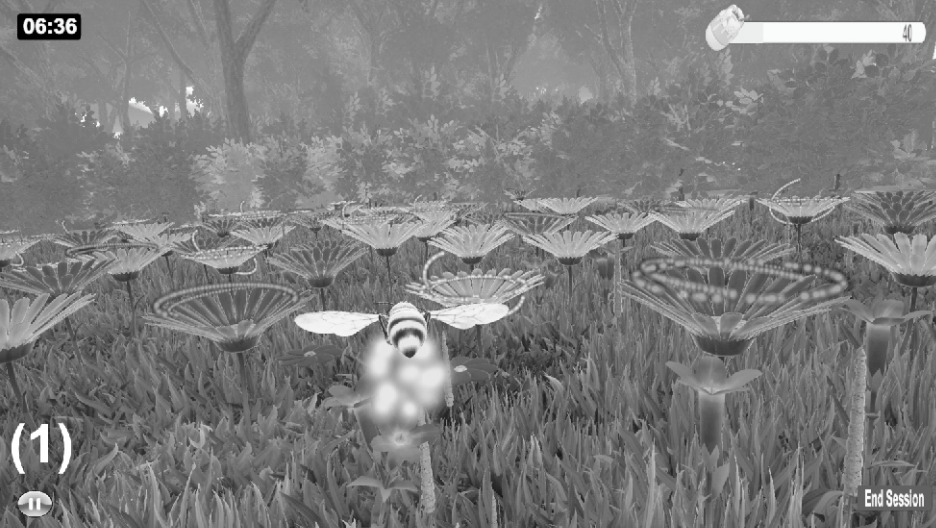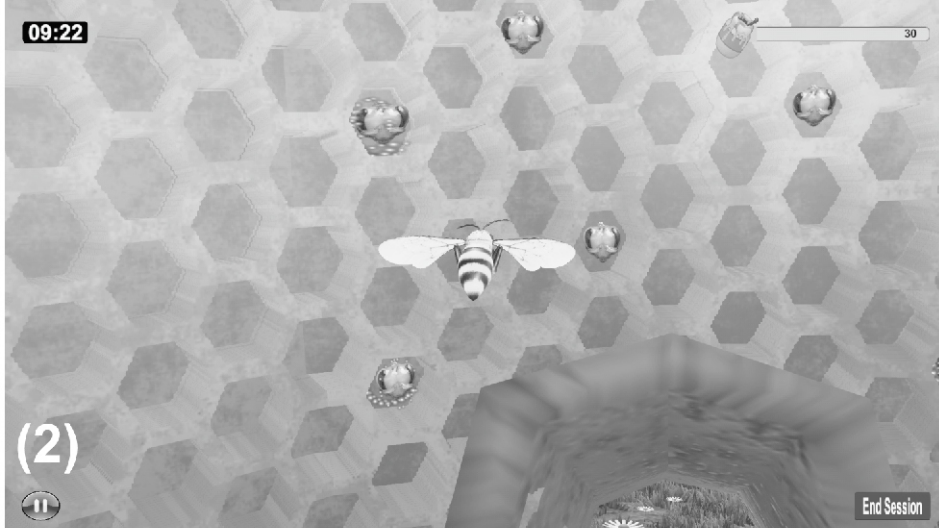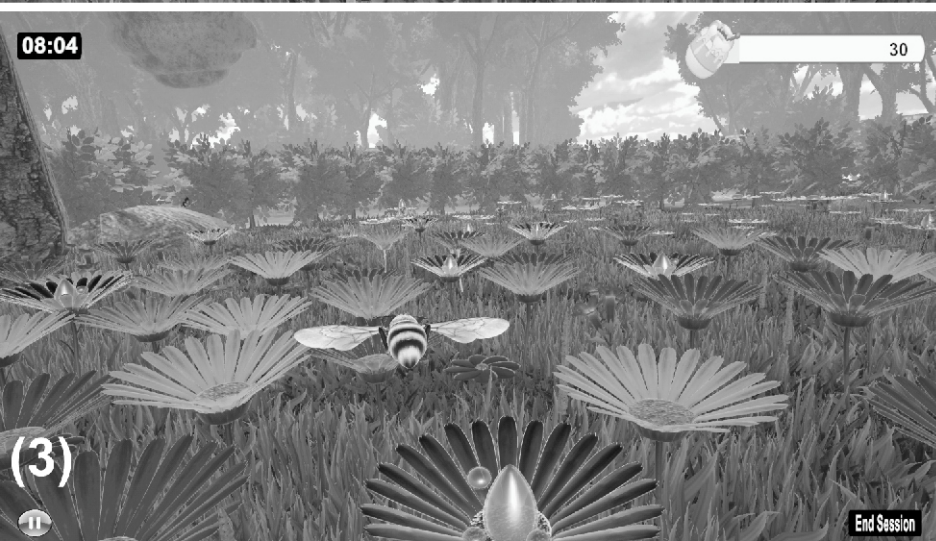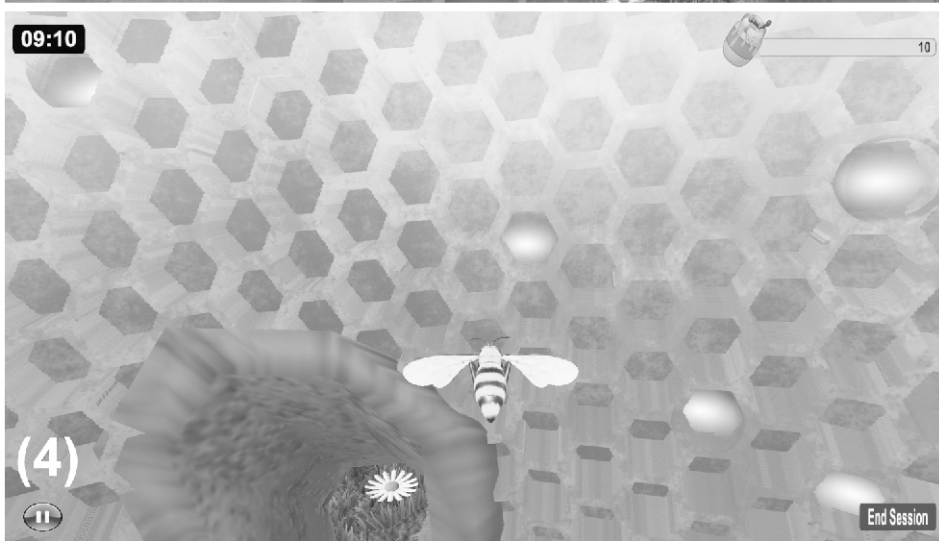

Supplement: Supplemental Information 2 — (1) Phase 1: Pollinating the flowers. (2) Phase 2: Feeding the larvae. (3) Phase 3: Collecting the nectar. (4) Phase 4: Drying the nectar. [file peerj-cs-09-1267-s002.pdf]

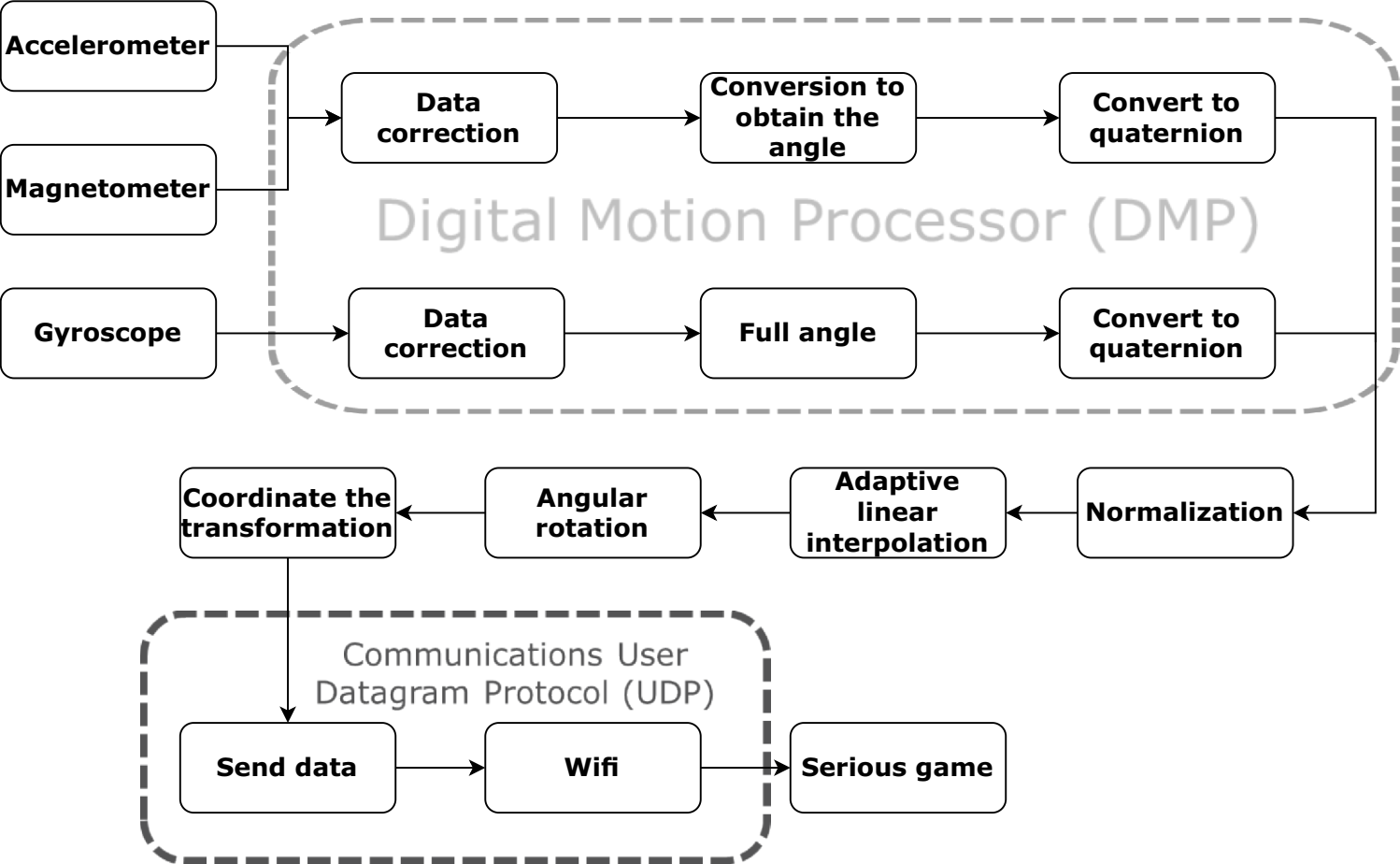

Supplement: Supplemental Information 3 [file peerj-cs-09-1267-s003.pdf]

<< RehaBEElitation>>

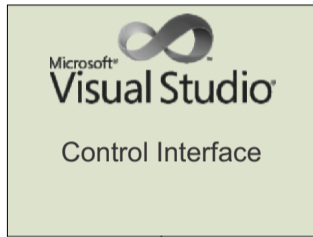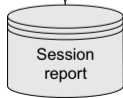

PostgreSQL

<< Serious Game>>

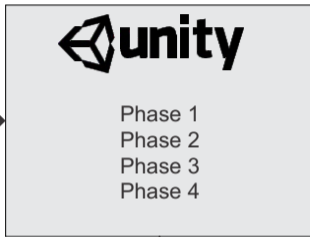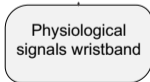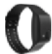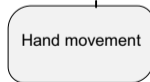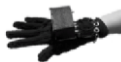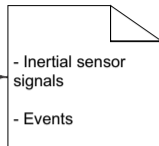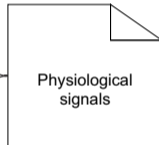

Supplement: Supplemental Information 6 [file peerj-cs-09-1267-s006.pdf]

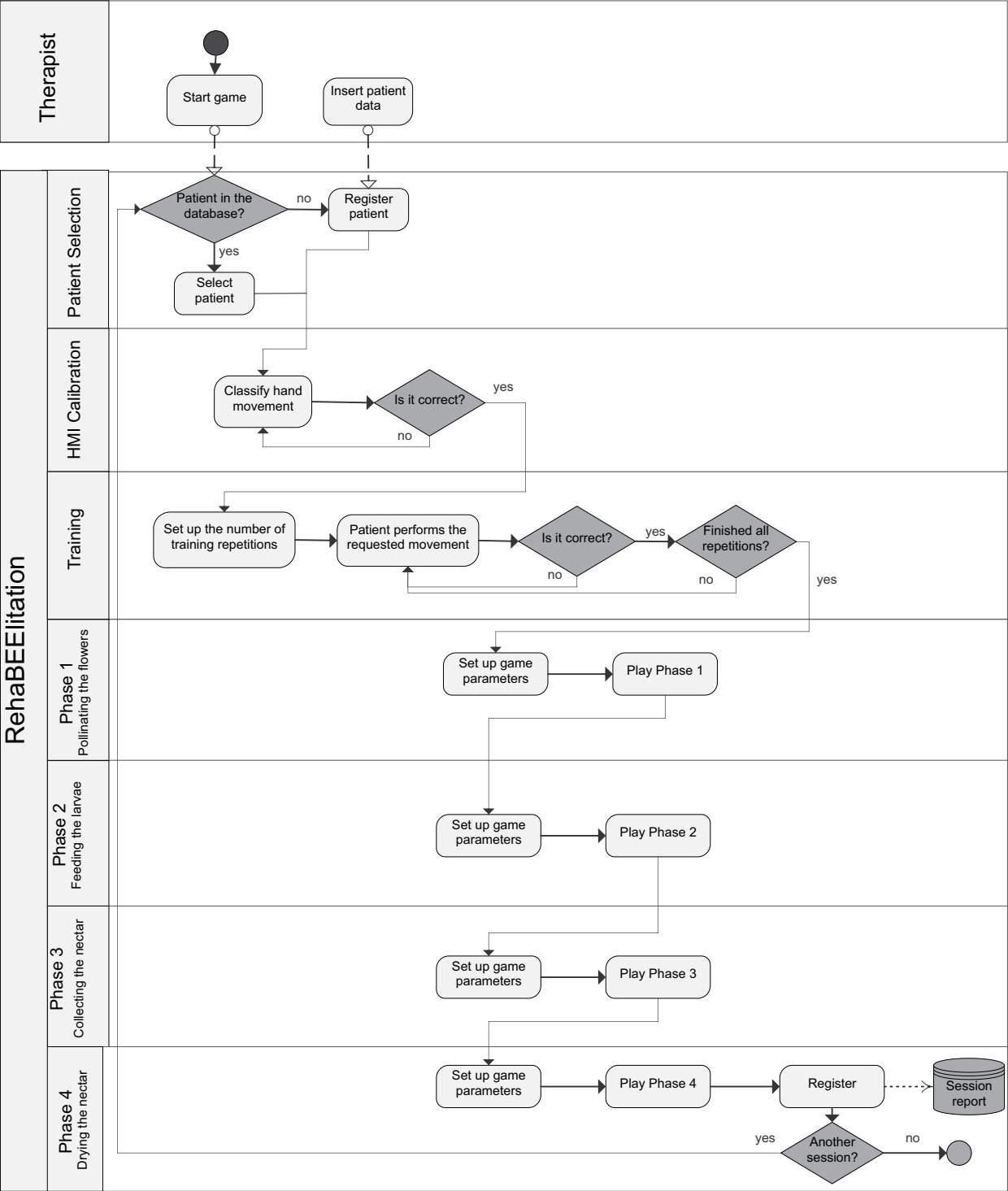

Supplement: Supplemental Information 7 [file peerj-cs-09-1267-s007.pdf]
