# Supplementary material for: RehaBEElitation: the architecture and organization of a serious game to evaluate motor signs in Parkinson’s disease
Source: PeerJ Comput Sci. 2023 Mar 15;9:e1267. doi: 10.7717/peerj-cs.1267 (PMC10280492; doi:10.7717/peerj-cs.1267)
Supplement: Supplemental Information 4 [file peerj-cs-09-1267-s004.docx]

**Attachment 4.** Relationship between states, hand movements and bee movements in the game.

| **State** | **HMI Movement** | **Bee Movement** |
| --- | --- | --- |
| 1 | Resting | Moves ahead |
| 2 | Extension – Open hand | Up |
| 3 | Flexion – Open hand | Down |
| 4 | Abduction – Open hand | To the left |
| 5 | Adduction – Open hand | To the right |
| 6 | Finger tapping | Collect nectar |
| 7 | Extension – Closed hand | Up and carrying pollen |
| 8 | Flexion – Closed hand | Down and carrying pollen |
| 9 | Abduction – Closed hand | To the left and carrying pollen |
| 10 | Adduction – Closed hand | To the right and carrying pollen |
| 11 | Pronation | Increases wing speed |
| 12 | Supination | Decreases wing speed |
