# Supplementary material for: RehaBEElitation: the architecture and organization of a serious game to evaluate motor signs in Parkinson’s disease
Source: PeerJ Comput Sci. 2023 Mar 15;9:e1267. doi: 10.7717/peerj-cs.1267 (PMC10280492; doi:10.7717/peerj-cs.1267)
Supplement: Supplemental Information 5 [file peerj-cs-09-1267-s005.docx]

**Attachment 5.** Events defined in the game.

| **Event identification** | **Description of the event** | **Observations** |
| --- | --- | --- |
| 1 | Bee standing still without moving in the scenario | In phases 2 and 4 |
| 2 | Bee moving forward | In phases 1 and 3 |
| 3 | Collects pollen | Movement of closing the hand |
| 4 | Deposits pollen | Movement of opening the hand |
| 5 | Bee moves to the right | Player with open hand |
| 6 | Bee moves to the right | Player with closed hand |
| 7 | Bee moves to the left | Player with open hand |
| 8 | Bee moves to the left | Player with closed hand |
| 9 | Bee moves up | Player with open hand |
| 10 | Bee moves up | Player with closed hand |
| 11 | Bee moves down | Player with open hand |
| 12 | Bee moves down | Player with closed hand |
| 13 | Collects nectar | Finger tapping movement |
| 14 | Dries nectar | Supination movement |
| 15 | Dries nectar | Pronation movement |
| 16 | Releases/deposits pollen at the wrong time (player's failure) | Negative feedback |
| 17 | Visual and sound feedback when the player reaches the target | Positive feedback |
| 18 | Sound feedback when the player scores a point | Positive feedback |
| 19 | Visual and sound feedback when the player doesn't move in the game for 30 seconds | Reinforcement feedback |
| 20 | Visual and sound feedback when the player doesn't score a point for one minute and 20 seconds | Reinforcement feedback |
| 21 | Visual and sound feedback when the game time is up (game over) | Negative feedback |
| 22 | Visual and sound feedback when the player completes the level | Positive feedback |
| 23 | Phase is paused | Glove data are not recorded |
