# Supplementary material for: RehaBEElitation: the architecture and organization of a serious game to evaluate motor signs in Parkinson’s disease
Source: PeerJ Comput Sci. 2023 Mar 15;9:e1267. doi: 10.7717/peerj-cs.1267 (PMC10280492; doi:10.7717/peerj-cs.1267)
Supplement: Supplemental Information 8 [file peerj-cs-09-1267-s008.docx]

**Attachment 8.** Main characteristics of the participants of the study.

| **HEALTHY GROUP** | | **EXPERIMENTAL GROUP** | | | | | |
| --- | --- | --- | --- | --- | --- | --- | --- |
|  |  | **PD-OFF** | | | | **PD-ON** | |
| **Id** | **Age** | **Id** | **Age** | **MDS-UPDRS III** | **Hoehn and Yahr** | **MDS-UPDRS III** | **Hoehn and Yahr** |
| 79 | 82 | 65 | 57 | 61 | 3 | 25 | 2 |
| 72 | 64 | 53 | 80 | 49 | 2 | 25 | 2 |
| 59 | 80 | 25 | 64 | 58 | 2 | 26 | 2 |
| 37 | 66 | 43 | 77 | 44 | 2 | 31 | 2 |
| 76 | 51 | 68 | 59 | 47 | 3 | 24 | 2 |
| 75 | 56 | 24 | 64 | 48 | 2 | 39 | 2 |
| 46 | 77 | 28 | 70 | 41 | 2 | 20 | 2 |
| 78 | 62 | 66 | 73 | 45 | 3 | 42 | 3 |
| 69 | 60 | 40 | 60 | 29 | 2 | 12 | 1 |
| 80 | 80 | 60 | 66 | 29 | 1 | 16 | 1 |
| 71 | 69 | 39 | 69 | 70 | 3 | 37 | 2 |
| 73 | 59 | 36 | 60 | 54 | 2 | 31 | 2 |
| 77 | 62 | 49 | 50 | 59 | 4 | 35 | 3 |
| 74 | 55 | 29 | 68 | 52 | 2 | 38 | 2 |
| 35 | 71 | 26 | 77 | 38 | 1 | 11 | 1 |
